# Determining the Molecular Pathways Underlying the Protective Effect of Non-Steroidal Anti-Inflammatory Drugs for Alzheimer's Disease: A Bioinformatics Approach

**DOI:** 10.1016/j.csbj.2016.10.003

**Published:** 2016-10-29

**Authors:** Alejo J Nevado-Holgado, Simon Lovestone

**Affiliations:** Department of Psychiatry, University of Oxford, Warneford Hospital, Oxford, OX3 7JX, UK

**Keywords:** AD, Alzheimer's Disease, GWAS, Genome-wide association study, NSAID, Non-steroid anti-inflammatory drugs, KEGG, Kyoto Encyclopedia of Genes and Genomes, Alzheimer's disease, NSAID, Inflammation, Fuzzy logic, Ribosome

## Abstract

Alzheimer's disease (AD) represents a substantial unmet need, due to increasing prevalence in an ageing society and the absence of a disease modifying therapy. Epidemiological evidence shows a protective effect of non steroidal anti inflammatory (NSAID) drugs, and genome wide association studies (GWAS) show consistent linkage to inflammatory pathways; both observations suggesting anti-inflammatory compounds might be effective in AD therapy although clinical trials to date have not been positive.

In this study, we use pathway enrichment and fuzzy logic to identify pathways (KEGG database) simultaneously affected in both AD and by NSAIDs (Sulindac, Piroxicam, Paracetamol, Naproxen, Nabumetone, Ketoprofen, Diclofenac and Aspirin). Gene expression signatures were derived for disease from both blood (n = 344) and post-mortem brain (n = 690), and for drugs from immortalised human cell lines exposed to drugs of interest as part of the Connectivity Map platform. Using this novel approach to combine datasets we find striking overlap between AD gene expression in blood and NSAID induced changes in KEGG pathways of Ribosome and Oxidative Phosphorylation. No overlap was found in non NSAID comparison drugs. In brain we find little such overlap, although Oxidative Phosphorylation approaches our pre-specified significance level.

These findings suggest that NSAIDs might have a mode of action beyond inflammation and moreover that their therapeutic effects might be mediated in particular by alteration of Oxidative Phosphorylation and possibly the Ribosome pathway. Mining of such datasets might prove increasingly productive as they increase in size and richness.

## 1 Introduction

Alzheimer's disease (AD) is one of the largest single unmet medical needs today, due partly to the increasing numbers of cases in an ageing society [1], [2], partly due to the absence of any disease modifying therapy [3] and mostly due to the very high cost of caring for people with dementia. It is known that environmental factors can alter risk of AD, such as mid-life obesity or history of diabetes [4], [5], [6]. With respect to medications, for more than three decades, epidemiological studies have shown that users of non-steroidal anti-inflammatory drugs (NSAID) are less likely to develop AD later in life, an effect observed most strongly after prolonged drug use [7], [8], [9]. More recent observational studies have largely replicated this finding [10], [11], [12], [13], [14], [15], but to date there has been no translation of this observation into a usable therapy in randomised trials [16], [17], [18].

This failure of translation may be due to trials-related factors such as interventional studies being conducted too late in disease process or to disease-related factors such as AD being of mixed aetio-pathogenesis and NSAIDs being effective only in some, as yet unknown, sub-group of disease. Or, conceivably at least, it might be due to compound-related factors. That is, it might be that only some NSAIDs might have efficacy in reducing risk of AD and that selective effectivity might be due to a mode of action beyond the obvious and shared action of this class of compound. To explain and circumvent this failure in translation, studies have attempted to investigate the mechanism through which NSAIDs lower AD risk. Some studies link its benefit to the capacity of NSAIDs to suppress microglia activation due to its anti-inflammatory effect [19], [20], [21]. However, aspirin is an example of an anti-inflammatory with a prescription profile very similar to NSAIDs [22], [23], that does not appear to lower AD risk [10], [24]. Whilst it remains likely that it is the anti-inflammatory effect of the NSAIDs that reduces risk of AD, it is possible there are other mechanisms of action in at least some of this class of compound, and some of these non-inflammatory mechanisms might affect disease risk. For example, some studies suggest that NSAIDs may reduce AD risk through direct effects on the formation of Aβ, a core pathogenic event in AD [20], [25], possibly through COX inhibition [26] or through its effect on gamma-secretase [27], [28]. However, others have concluded that the relationship between NSAIDs use and AD risk is independent of the effect that these drugs have on Aβ [29], [30]. The mechanism of effect of the NSAIDs in reducing risk of AD therefore remains uncertain, hampering attempts to translate the epidemiological finding to an effective therapy.

Here we use gene expression studies to identify molecular pathways affected by a range of NSAIDs, and use enrichment analysis and fuzzy logic to determine overlap with gene expression studies in AD in man. To be able to link results from gene expression studies with molecular effects of NSAID drugs, we analyse RNA arrays of in-vitro human cells exposed to a range of NSAID molecules. The degree of dysregulation of each gene in multiple assays (i.e. gene expression from either blood, brain and/or cells exposed to drugs) is combined to detect whether any pathway is more dysregulated across assays than what should be expected from chance. These studies suggest that the NSAIDs might induce changes in gene expression in pathways not obviously related to inflammation but ones which might affect AD risk, including in ribosome function and oxidative stress. These findings suggest novel approaches to therapeutic development in dementia.

## 2 Methods

**Fig. 1:**
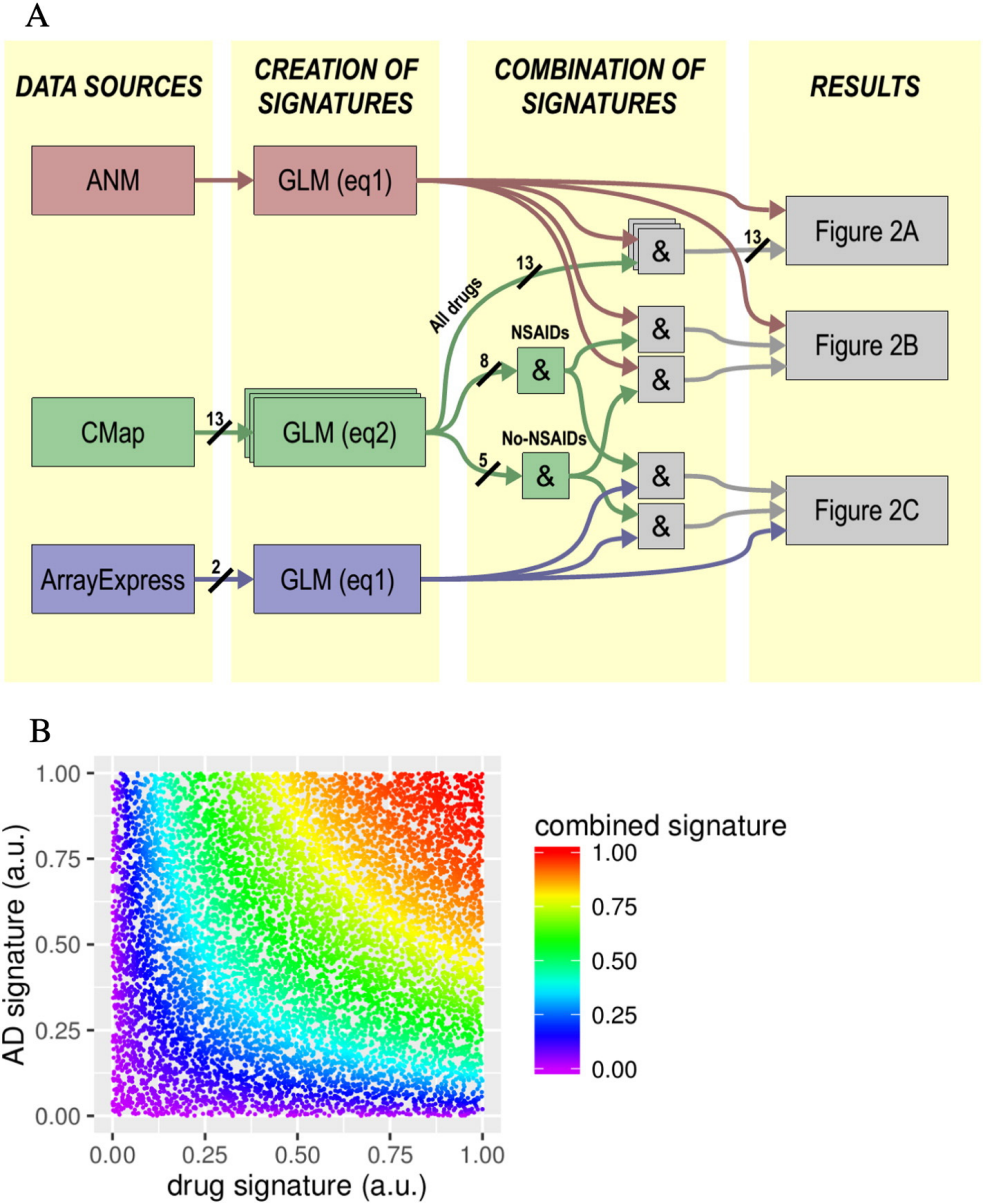
Analysis methods: (A) Flowchart summarising methods. Background yellow boxes represent the different stages, from left to right: data sources (methods Section 2.1); generation of signatures (methods 2.2, 2.3); combination of signatures (methods Section 2.4); and results from pathway enrichment (methods Section 2.5) and as shown in the result figures. When arrows represent more than one dataset or more than one signature, a slash and a number identify the number of datasets/signatures involved (e.g. 13 datasets were extracted from CMap and analysed with the GLM of Eq. (2)). (B) Example of combination of signatures. Each one of the dots in the figure represent one of the genes sampled in AddNeuroMed and CMap. For each dot, its Y position, X position and colour represent, respectively, its level on truth in the AD signature, Diclofenac signature and combined signature. The figure was created with the product fuzzy gate described in the main text, which gives high levels of truth only to the genes that had high values in all original signatures.

In order to identify molecular pathways induced by NSAIDs that might affect susceptibility to AD, we sought genes dysregulated by both AD and NSAIDs to a larger degree than that expected by chance. In order to identify such pathways we developed and applied a three-step method: first we derive a genetic signature of AD from gene expression studies and for each of a number of drugs from experimental studies in vitro. This genetic signature is a value in the [0–1] range per gene, where the value is proportional to the dysregulation of that gene in AD patients, or in cells exposed to the drug under consideration. Secondly, using fuzzy logic we combine these genetic signatures of drug and disease, looking in effect for genes common to both. In practice, this second step derives a new [0–1] number per gene, which reaches high values only for genes that had a value close to 1 in both the original signatures (see Fig. 1). Thirdly and finally, we estimate whether these combined signatures enrich any given pathway more than that expected by chance. Specifically, this third steps identifies whether the [0–1] numbers derived in step two are significantly altered in any given molecular pathway in the KEGG pathway set.

### 2.1 Data Source

Three different publicly available datasets, one from blood and two from post-mortem brain, were used to identify a gene expression signature of AD (see Supplementary Fig. 3). The first dataset was AddNeuroMed (GeneExpressionOmnibus: GSE63063), a cross-European cohort studies with whole genome expression data from blood from 200 AD patients and 192 controls [31], [32]. The second dataset was from post-mortem pre-frontal cortex of 129 AD patients and 101 controls [33] Finally, the third dataset was from post-mortem hippocampus of 80 ADs and 173 controls [34]; ArrayExpress: E-GEOD-48350). In order to derive a signature of AD, data was log-transformed independently in each dataset. With these three datasets we derive two different signatures. From the AddNeuroMed we derive a genetic signature describing the dysregulation of genes in blood of AD patients. The other two datasources are merged into a single dataset, from which we derive a second signature that reports the dysregulation of genes in post-mortem brain of AD patients.

In addition to the three human datasets used to determine gene expression signatures from AD, we used the Connectivity-Map (CMap) to determine the gene expression signatures of a range of different NSAIDs and a comparison group of commonly prescribed, non-NSAID compounds (8 NSAIDs and 5 non-NSAIDs). The CMap study is a publically accessible resource of gene expression derived from immortalised cell lines (MCF7, PC3 and HL60 cells), exposed to a large range of compounds at different concentrations [35], [36]. For each investigated drug, all available data was used including that from all cell lines, all drug concentrations and the technical variables including those related to expression analysis (vendor and scanner). With these 13 CMap datasets, we derive 13 genetic signatures, each signature representing the dysregulation of gene expression for each drug. These 13 signatures are later combined with each other and with the 2 AD signatures (see Section 2.4).

### 2.2 Genetic Signatures for AD

The genetic signature of each perturbation (i.e. each given drug and/or AD) represents the level of dysregulation in gene expression generated by the perturbation under consideration. In the case of AD, this is calculated with a logistic Generalised Linear Model (GLM) that controls for age, gender and data source (i.e. dataset of origin and centre where the blood/brain sample was collected). The equation of the GLM, in R formula syntax, is:

(1) ad~rna+age∗source+gender∗source

where “ad” is a binary variable representing AD status (i.e. subject is either AD patient or control), rna is the RNA expression level, age is subject age, source is sample dataset and/or sample of origin, and gender is the gender of each subject. The so-called signature is then 1 minus the p-values associated to the variable “rna” in the aforementioned GLM model.

The subtraction to 1 is applied for the signature to behave as a “value of truth” of fuzzy logic, as fuzzy operations are used to combine signatures later in the overall analysis of this study. Lower values in the signature for a given gene, will indicate that the expression of this gene is unlikely to be altered in AD. Higher values, however, will indicate an increasing likelihood that expression of the gene is dysregulated in the disease state.

We highlight that we derive two different AD signatures. One of the signatures represents the gene dysregulation in AD blood, and it is obtained by applied the linear model to the AddNeuroMed dataset. The other signature represents the genetic dysregulation in post-mortem AD brain, and it is obtained by applying the linear model to the dataset resulting from merging both ArrayExpress cohorts.

### 2.3 Genetic Signatures for Drugs

Similar to the genetic signature calculated for AD, the signature for each of the investigated drugs is also derived with a logistic GLM, which controls for all the covariants that may influence gene expression. For drugs, these variables are each type of cell line, batch, vendor, concentration and scanner, forming the following GLM equation in R formula syntax:

(2) vehicle~rna+cell+batch+vendor+concentration+scanner

where “vehicle” is a binary variable representing drug status (i.e. whether the sampled cell culture was exposed to the drug or not). The so-called signature is again 1 minus the p-values associated to the variable “rna” in the aforementioned GLM model.

This linear model is applied independently to each one of the 13 CMap datasets. In each case, we obtain a signature representing the genetic dysregulation present in the cells exposed to a given drug (8 NSAIDs and 5 non-NSAIDs).

### 2.4 Combination of Signatures

Fuzzy logic is an extension of Boolean logic where the binary values of truth (i.e. 0 for false, 1 for true) are transformed into a continuous scale from 0 to 1, which then represent intermediate values of truth or certainty (e.g. 0.0 for ‘fully false’, 0.1 for ‘most certainly false’, 0.5 for ‘unknown’, or 0.7 for ‘probably truth’). Logic operations (such as AND, OR, NOT) are also extended to the full [0,1] range, allowing the combination of fuzzy variables into more complex logical concepts (e.g. ‘a AND b’, where ‘a’ and ‘b’ are fuzzy variables with values of truth between 0 and 1).

Mirroring the techniques applied in fuzzy logic, we combine the derived signatures with a product operation. Given a number of to-be-combined signatures, denoted “s(g,p)” for gene “g” and perturbation “p”, the combined signature is:

(3) $$s_{I}\left(g\right)=\prod_{i\in I}s\left(g,i\right)$$

where “sI(g)” represents the combined signature, and “I” represents the set of signatures to combine. For instance, the combined signature of AD from blood and Diclofenac (an NSAID) would be:

(4) $$s_{\mathrm{AD}\cdot \mathrm{Diclofenac}}\left(g\right)=s\left(g,\mathrm{AD}\right)\cdot s\left(g,\mathrm{Diclofenac}\right)$$

The actual values of this example are represented in Fig. 1. For genes to obtain a high fuzzy “level of truth” in the combined signature, the gene needs to have a high value in both the AD and the Diclofenac signatures. The reason is that the product rule implements the fuzzy version of the boolean operation AND. Therefore, the genes that will obtain the highest values of truth in the combined signature will be those that had high value of truth in all original signature simultaneously.

In most instances Eq. (3) is used to combine single pairs of signatures into one. However, in two instances, Eq. (3) is used to combine groups of more than 2 signatures. Namely, in one instance we combine all 8 NSAIDs into a single signature, which is later combined again with the signatures from AD blood and AD brain separately (see Fig. 1A). In another instance, we combine all 5 non-NSAID drugs into a single signature, which is later combined with this one from AD blood and from AD brain.

### 2.5 Pathway Analysis

Pathway enrichment is implemented through the Kolmogorov–Smirnov (KS) non-parametric test. This test has two main advantages in comparison with the binomial test often used in pathway analysis. First, in the binomial test the genes that are going to be considered as “positive” (i.e. significantly dysregulated) are commonly defined as those genes that pass a multiple comparison corrected p-value threshold. However, our method delivers “values of truth”, which may not be interpretable as p-values themselves. The KS test does not require p-values, but rather a list of numbers per genes which bears information in their rank (e.g. genes with higher values are more dysregulated than genes with lower values). Secondly, KS makes use of the whole distribution of values per gene (these can be a p-value per gene or, in our case, a fuzzy level of truth per gene), while the binomial test down-samples these gene values to a binary variable before running the actual statistical test.

Given a genetic signature, we apply the KS test to each KEGG pathway independently. With KS we compare the signature values of all those genes included in the pathway against the signature value of all the genes not included in the pathway. The obtained p-value indicates whether the signature levels of truth of the genes that are present in that pathway are significantly larger than the values typically observed in all other pathways.

## 3 Results

In order to determine gene expression signatures that overlap between AD and NSAIDs we used a three step process – first determining, from patient samples from blood and brain, the gene expression signature of AD [31], [32], [33], [34] as well as the gene expression signature induced by NSAIDs in the Connectivity Map (CMap) database [35], [36]; second determining the overlap in pathways induced by these drugs and that found in AD; and thirdly estimating the overlap expected by chance alone. The drug signatures were calculated for the most commonly prescribed NSAID drugs included in CMap study: Sulindac, Nabumetone, Ketoprofen, Piroxicam, Naproxen, Diclofenac, Paracetamol and Aspirin (Acetylsalicylic Acid). For comparison, we also calculated the signature of a number of non anti-inflammatory drugs, including both drugs indicated for neuropsychiatric conditions and other commonly prescribed compounds: Carbamazepine, Amitriptyline, Apomorphine, Ramipril and Simvastatin. As gene expression signatures in disease in blood and in brain may reflect different aspects of the disease process, we first performed an analysis for blood expression signature and then for brain.

### 3.1 Individual Drugs and AD Blood

For each drug, we combined its gene expression signature with that derived from an analysis of gene expression in blood in AD (AddNeuroMed, 105 AD patients and 114 controls [31], [32]), after which KS pathway enrichment was applied to all the 223 KEGG pathways that had genes sampled in both AD blood and CMap.

**Fig. 2:**
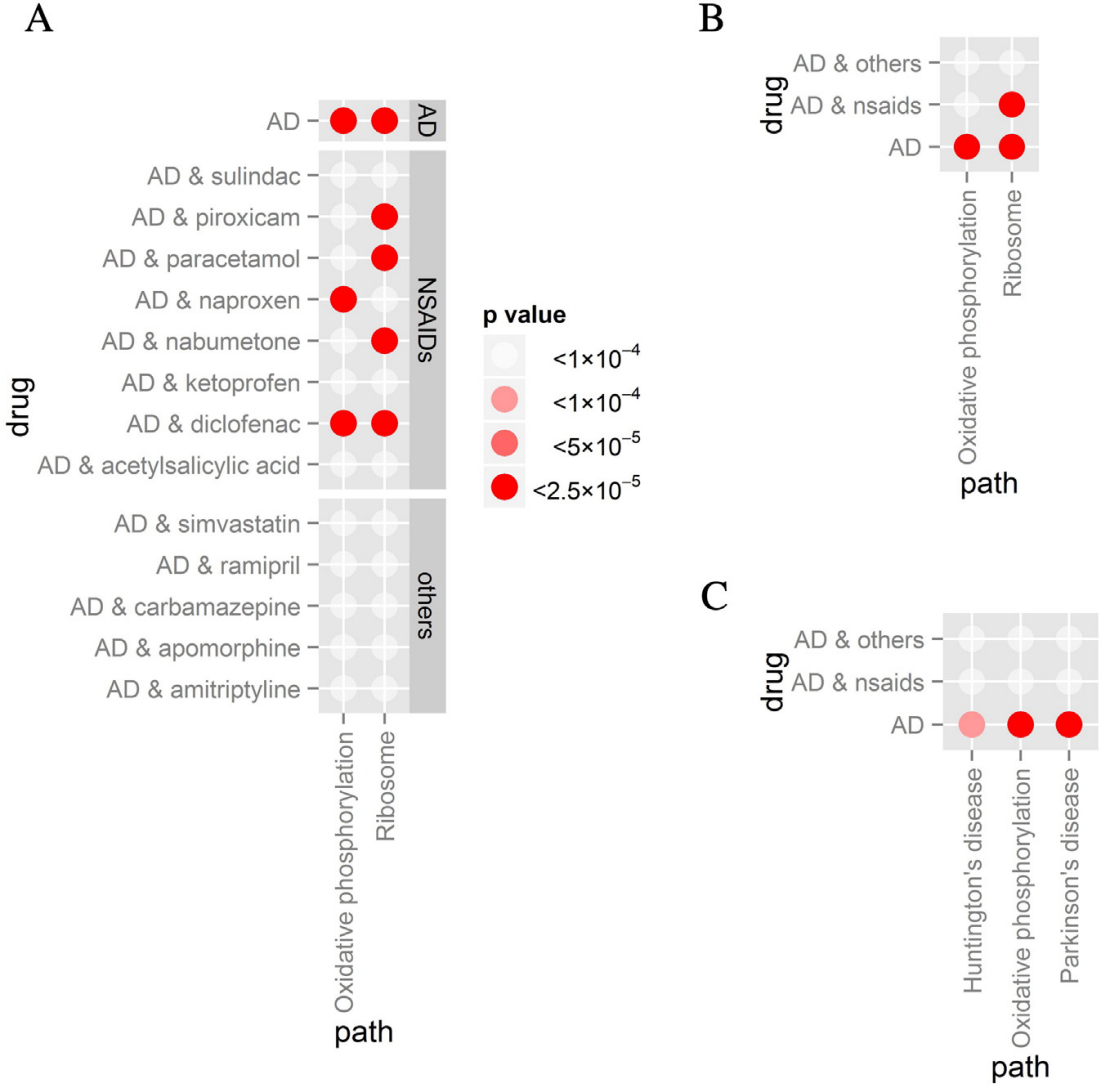
Statistical results: The figures show the results from the KS pathway enrichment applied to the different genetic signatures. (A) Results when pathway enrichment is applied to either the original blood AD signature (first row in the figure), or to the signatures of AD combined with different drugs (remaining 13 rows). (B) Results when enrichment is applied to the signatures combining blood AD with different drug groups. (C) Results from pathway enrichment when applied to the signatures combining brain AD with different drug groups. (A, B & C) In all cases, each dot represents the p-value for a given signature (Y-axis) and on a given pathway (X-axis), while its colour represents the obtained p-value. Only pathways that had a p-value below 0.0001 in any of the signatures are shown. The IDs of the KEGG pathways are: Oxidative phosphorylation 00190; Ribosome 03010; Huntington's disease 05016; Parkinson's disease 05012.

Using an uncorrected p-value threshold of 0.0001, the KEGG pathway ‘Ribosome’ (ID 03010) was strongly significant for 4 out of 8 NSAIDs (Piroxicam p = 5.36 × 10^–^^9^, Paracetamol p = 3.12 × 10^–^^6^, Nabumetone p = 1.78 × 10^–^^6^ and Diclofenac p = 1.40 × 10^–^^9^), while the KEGG pathway ‘Oxidative Phosphorylation’ (ID 00190) was strongly significant for two (Naproxen p = 1.21 × 10^–^^5^ and Diclofenac p = 1.85 × 10^–^^5^, see Supplementary Table 1 and Fig. 2A). However, none of the five non-NSAIDs showed any pathway with significance below 0.0001. In the case of the signature of AD alone (i.e. not combined with the signature of any of the drugs), both Ribosome (p = 4.66 × 10^–^^7^) and Oxidative Phosphorylation (p = 7.28 × 10^–^^9^) had a p-value below this threshold. No other pathways had a p-value below 0.0001 for any of the signatures. When calculating the effect size of the linear models (Eqs. (1), (2)), the direction of effect of both AD tissue and NSAID drugs was more frequently negative (lower expression than controls), while direction of effect of non-NSAID drugs was more frequently positive (higher expression than controls) for the significant pathways (see Supplementary Fig. 4).

### 3.2 Grouping Drugs and AD Blood

Studies investigating the epidemiological relationship between NSAIDs and AD most often classify all NSAIDs into a single category, rather than investigating each drug independently [7], [8], [9]. To facilitate the interpretation of our results when compared to those of the epidemiological studies, we therefore used our method to combine the signature of all 8 NSAID drugs into a single signature. This signature was then combined with that from the AD blood dataset. In both cases (i.e. when combining multiple drugs into a single signature, and when further combining this one with that from AD), we used the same product function described in methods (Eq. (3)). Again, the Ribosome pathway was the most highly significant, with a p-value that would survive multiple comparisons correction (p = 7.38 × 10^-7^, see Supplementary Table 2 and Fig. 2B). For comparison, we also repeated the analysis for the aforementioned non-NSAID drugs, first combining them all into a single signature, and then further combining this one with AD's using the product function (Eq. (3)). However, in the case of non-NSAIDs, no pathway showed a p-value below 0.0001 (see Supplementary Table 2).

### 3.3 Grouping Drugs and AD Brain

The method described in Section 3.2 was further applied to the AD signature obtained from post-mortem brains (ArrayExpress E-GEOD-44770 and E-GEOD-48350, totalling 209 ADs and 274 controls). We found 227 KEGG pathways had genes sampled in both AD brain and CMap but when enrichment was applied to the disease signature alone the three pathways with p-value below 0.0001 were Huntington's Disease (p = 4.66 × 10^–^^7^), Parkinson's Disease (p = 4.66 × 10^–^^7^) and Oxidative Phosphorylation (p = 4.66 × 10^–^^7^, see Supplementary Table 3 and Fig. 2C). No pathways were significant below p-value 0.0001 when the AD signature was combined with either NSAIDs (Huntington's Disease p = 1.26 × 10^–^^4^, Parkinson's Disease p = 2.95 × 10^–^^4^, Oxidative Phosphorylation p = 1.53 × 10^–^^4^) or non-NSAIDs (Huntington's Disease 0.12, Parkinson's Disease 0.0058, Oxidative Phosphorylation 0.064) drug groups (see Supplementary Table 3) although all three combined AD and NSAID signatures approached our predermined p-value cut-off.

## 4 Discussion

As described in the introduction, it is widely replicated and accepted that prolonged NSAID medication correlates with a decreased risk of suffering AD later in life. This observation has contributed to the emergence of the inflammatory hypothesis of AD. While the more traditional amyloid hypothesis states that neuroinflammatorion is a downstream consequence of amyloid accumulation, the inflammatory hypothesis states that the immune system may in fact contribute and drive AD pathology [37]. However, despite considerable efforts, it has not been possible to translate the NSAID-AD observation and the inflammatory hypothesis into a disease-modifying therapy. Some of the failed NSAID trials used Ibuprofen [38], Naproxen [39], [40], [41] or Celecoxib [39], [40], [42], with all of them either showing no results when compared to placebo, or being discontinued due to cardiovascular or other side effects. Further insight into what is the molecular background where NSAID and AD may intersect would likely help on facilitating this translation to the clinic.

In this study, we used a three step process to explore whether any gene expression overlap exists between AD and NSAIDs in an attempt to determine whether there are effects of these drugs that might be relevant to disease that go beyond the generic or shared effects on inflammation. First, we derived gene expression signatures of AD from blood and from brain together with the gene expression signature induced by NSAIDs in the Connectivity Map (CMap) database; second, we determined the overlap in pathways induced by these drugs and those altered in AD; and thirdly, we estimated the overlap expected by chance alone. Comparing these signatures, we find that the Ribosome pathway, and to a lesser degree Oxidative Phosphorylation, are more often shared by both AD, at least in blood, and NSAIDs than that expected by chance (Fig. 2A and B). This association is not found for drugs other than NSAIDs, or when post-mortem AD brain samples are used instead of AD blood (Fig. 2A, B and C) although the finding for Oxidative Phosphorylation approaches a pre-specified significance level. Given that the method is fully data driven and based on gene expression, it could be applied in the future towards personalised medicine. Namely, genetic signatures could be derived from blood of specific AD patients, and/or from iPSC cells derived from a target patient and then exposed to a number of drugs [43], [44], [45]. The results would then be applicable to that specific patient, rather than to the undifferentiated population of all AD patients.

As described in methods, the fuzzy AND operand (Eq. (3)) promotes those genes whose “level of truth” in relation to expression is high simultaneously in all of the original signatures. For instance, if AD is combined with Diclofenac, a gene with a low value in AD but a high value in Diclofenac, will obtain a low combined level of truth. Only the genes with a level of truth close to 1 in both AD and Diclofenac, will also obtain a combined level of truth close to 1. Therefore, when pathway enrichment is applied to the combined signatures, the significant pathways are those that were strongly dysregulated in both AD patients and the CMap cells exposed to the drugs. Therefore, Fig. 2A and B indicate that genes of the Ribosome pathway are simultaneously perturbed by both AD and NSAIDs, to a degree larger than that expected from chance alone. This is unlikely to be an effect of the overall method itself, as Fig. 2A, B and C evidence that this level of simultaneous dysregulation is not observed in other, non-NSAID, drugs.

It is however important to emphasise that our method only attends to dysregulations in gene expression, without considering the direction of the dysregulation. This direction-free analysis was employed as we cannot predict whether a compound altering risk of disease should increase or decrease gene expression. It might be for example that a given gene is increased in AD due to a protective mechanism in response to disease, albeit one that was ultimately unsuccessful. In this case then if the protective effect of a given NSAID was mediated in part through this gene then one would predict an increase in gene expression in the experimental in vitro CMap data. On the other hand if a given gene was increased in AD because of its involvement in the pathological process then if the protective effect of a given NSAID was mediated through this gene, one would predict that in the CMap data, this gene would be down-regulated by that NSAID. Given this uncertainty regarding direction of effect, even for a given pathway where typically some genes increase and some decrease, pathway activity, then analysis that is agnostic to direction of effect is appropriate.

However, in contrast to blood, we found no pathway simultaneously dysregulated in both cell lines exposed to NSAIDs and post-mortem AD brain at our pre-specified level of significance (p < 1 × 10^–^^4^), although all three pathways, identified as dysregulated in AD brain come close to significance. The relative lack of significance in the post-mortem derived signature and CMap NSAID drugs might be due to a number of potential reasons, of which two stand out for us. First, the gene expression set from blood is derived from research participants relatively early in disease process whereas the brain signature is derived from end-stage disease. Although a brain expression set is, superficially at least, more relevant to a brain disease, this may well not be true. By end-stage disease, especially for a disease such as AD with a 10–20 year course from preclinical to end-stage, the expression of genes may no longer reflect primary aetiopathogenic process but a complex pattern of change derived from neurotoxicity, secondary effect of loss of neurons, protective mechanisms and end of life changes including agonal state and post-mortem artefact. Blood expression data on the other hand, although from a tissue remote from the site of disease, reflects early phase disease processes and is relatively free from the artefactual confounds that plague post-mortem study. The finding of overlap between gene signatures induced by NSAIDs and in blood early in disease process is particularly interesting given the increasing evidence that the protective effect of NSAIDs in relation to AD is also early in the disease process [14]. The second potential explanation for the failure to identify an overlap pathway in brain as we find in blood might be due to the site of action of the NSAIDs. Whilst it is commonly assumed that AD is a disorder exclusively of brain, this also may not be entirely true. It is possible that AD is a disease with a brain manifestation of a systemic change, that is that there are molecular changes that are systemic but that only cause a pathological process in brain, perhaps because neurons, as non-dividing long-lived cells are particularly sensitive to whatever pathways underlie this systemic process. The concept of a systemic component of AD has been widely postulated, including both inflammatory and non-inflammatory processes [35], [36], [37] and it is possible that the altered expression of genes in blood represents this systemic component and also possibly that the overlap in gene expression we find between blood and NSAIDs represents the protective actions of NSAIDs in relation to this systemic component.

The two KEGG pathways we observe overlapping between NSAIDs and AD blood gene expression are those of Oxidative Phosphorylation and Ribosome. Moreover, despite the overwhelming significance of the overlap in NSAID signatures and AD blood gene expression and the relative lack of overlap with AD brain expression, it is noteworthy that Oxidative Phosphorylation also approaches significance in NSAID to brain analyses. Oxidative stress has long been identified as a component of the many pathophysiological events of AD [49], [50] and has previously been postulated to underlie the association between AD and diseases of both metabolism and inflammation [51], [52]. Our findings add some weight to this hypothesis. The ribosomal pathway has not been associated with AD with the same frequency although ribosomal activity has been linked to AD in several studies [53], [54]. Moreover, ribosomal dysfunction in AD has been linked to RNA- and endoplasmatic reticulum- associated iron and oxidation [55]. More speculatively, ribosome mutations can cause familial Diamond-Blackfan anaemia [56] and increase risk of cancer [57], and both some cancers and some anaemias alter risk of AD from epidemiologic studies [47], [48], [49]. Our findings raise the possibility that if alterations in the Ribosome pathway are mediating this risk then an effect of NSAIDs on this pathway might in part explain their relative protective effect on AD.

There are obvious limitations to our study. Predominant amongst these is the relative paucity of data pertaining to altered gene expression in AD. We identified only one study of expression of genes in blood and the numbers of subjects in this and the brain datasets we used was small. The studies in brain will be subject to the confounds of late disease, agonal state and post-mortem change as discussed above. Moreover, both blood and brain studies are aggregates of many cell types and the gene expression most relevant to disease might be present in only one cell type – in brain, in neurons for example. In addition, the drug data (CMap) comes from non-CNS cells, which may have a different gene expression when exposed to NSAIDs. Some of these limitations are intrinsic to the field – pre-mortem gene expression studies in brain at scale are unlikely ever to be conducted for example. However, others will be increasingly overcome with some of the large scale studies currently underway to map molecular pathways of disease in ever larger datasets. Similarly, the CMap dataset is currently being enhanced in size and depth, which includes using a wide variety of cell lines, some of them from the CNS. Our study indicates that mining such datasets, not only independently but across different types of data, might be highly productive.

In conclusion, we have used a novel approach to test the hypothesis that NSAIDs might affect risk of disease not only through generic and known effects in suppressing inflammation but also through previously unrecognised molecular actions. To do this we have used fuzzy logic to combine gene expression changes induced by both disease and drug and find, in blood in particular, an overlap suggesting that one action of NSAIDs that might function to reduce risk of disease is on the KEGG pathways of Oxidative Phosphorylation and Ribosome. Further use of such bioinformatics approaches might be productive in identifying novel targets for therapy in this otherwise untreatable disorder.

## Appendix A Supplementary data

Supplementary material. Image 1
