# Supplementary material for: Determining the Molecular Pathways Underlying the Protective Effect of Non-Steroidal Anti-Inflammatory Drugs for Alzheimer's Disease: A Bioinformatics Approach
Source: Comput Struct Biotechnol J. 2016 Oct 29;15:1–7. doi: 10.1016/j.csbj.2016.10.003 (PMC5109283; doi:10.1016/j.csbj.2016.10.003)
Supplement: Supplementary file 1 — Supplementary material. [file mmc1.pdf]

**Appendix: Supplementary figures tables**

| | | Oxidative phosphorylation (ID 00190) | Ribosome (ID 03010) |
|---|---|---|---|
| p-values | AD | $7.28 \times 10^{-9}$ | $4.66 \times 10^{-7}$ |
| | AD & sulindac | 0.040 | 0.49 |
| | AD & piroxicam | $1.58 \times 10^{-4}$ | $5.36 \times 10^{-9}$ |
| | AD & paracetamol | 0.031 | $3.12 \times 10^{-6}$ |
| | AD & naproxen | $1.21 \times 10^{-5}$ | 0.30 |
| | AD & nabumetone | 0.49 | $1.78 \times 10^{-6}$ |
| | AD & ketoprofen | 0.0072 | 0.0076 |
| | AD & diclofenac | $1.85 \times 10^{-5}$ | $1.40 \times 10^{-9}$ |
| | AD & aspirin | 0.0022 | 0.0027 |
| | AD & simvastatin | 0.66 | 0.087 |
| | AD & ramipril | 0.36 | 0.49 |
| | AD & carbamazepine | $2.80 \times 10^{-4}$ | 0.0052 |
| | AD & apomorphine | 0.51 | $2.45 \times 10^{-4}$ |
| | AD & amitriptyline | $7.48 \times 10^{-4}$ | 0.96 |
| KS statistics | AD | 0.37 | 0.34 |
| | AD & sulindac | 0.15 | 0.073 |
| | AD & piroxicam | 0.25 | 0.38 |
| | AD & paracetamol | 0.16 | 0.31 |
| | AD & naproxen | 0.29 | 0.097 |
| | AD & nabumetone | 0.072 | 0.32 |
| | AD & ketoprofen | 0.19 | 0.19 |
| | AD & diclofenac | 0.28 | 0.37 |
| | AD & aspirin | 0.21 | 0.21 |
| | AD & simvastatin | 0.054 | 0.14 |
| | AD & ramipril | 0.085 | 0.074 |
| | AD & carbamazepine | 0.24 | 0.20 |
| | AD & apomorphine | 0.069 | 0.25 |
| | AD & amitriptyline | 0.22 | 0.016 |

Table 1, individual drugs combined with blood AD:

Results from the KS pathway enrichment, when applied to either the original blood AD signature, or to the signatures of AD combined with different drugs. The first 14 rows list uncorrected p-values per signature, while the last 14 rows represent the values of the statistic obtained from the Kolmogorov-Smirnov test. Only pathways that had a p-value below 0.0001 in any of the signatures are shown (i.e. Ribosome and Oxydative Phosphorylation, last two columns). Direction of effects are shown in supplementary Figure 4.

| | | Oxidative phosphorylation (ID 00190) | Ribosome (ID 03010) |
|---|---|---|---|
| p-values | AD & others | 0.02 | 0.22 |
| | AD & NSAIDs | 0.01 | $7.38 \times 10^{-7}$ |
| | AD | $7.28 \times 10^{-9}$ | $4.66 \times 10^{-7}$ |
| KS statistics | AD & others | 0.17 | 0.11 |
| | AD & NSAIDs | 0.18 | 0.33 |
| | AD | 0.37 | 0.34 |

Table 2, drugs groups combined with blood AD:

Results from the KS-pathway enrichment when applied to the signatures combining blood AD with different drug groups. As in supplementary Table 1, the first 3 rows list uncorrected p-values per signature, while the last 3 rows represent the values of the statistic obtained from the Kolmogorov-Smirnov test. Only pathways that had a p-value below 0.0001 in any of the signatures are shown

| | | Huntington's disease (ID 05016) | Oxidative phosphorylation (ID 00190) | Parkinson's disease (ID 05012) |
|---|---|---|---|---|
| p-values | AD & others | 0.12 | 0.0058 | 0.064 |
| | AD & NSAIDs | $1.26 \times 10^{-4}$ | $1.53 \times 10^{-4}$ | $2.95 \times 10^{-4}$ |
| | AD | $4.66 \times 10^{-7}$ | $4.66 \times 10^{-7}$ | $4.66 \times 10^{-7}$ |
| KS statistics | AD & others | 0.090 | 0.18 | 0.13 |
| | AD & NSAIDs | 0.19 | 0.22 | 0.22 |
| | AD | 0.19 | 0.28 | 0.32 |

Table 3, drugs groups combined with post-mortem brain AD:

Results from the KS-pathway enrichment when applied to the signatures combining brain AD with different drug groups. As in supplementary Tables 1 and 2, the first 3 rows list uncorrected p-values per signature, while the last 3 rows represent the values of the statistic obtained from the Kolmogorov-Smirnov test. Only pathways that had a p-value below 0.0001 in any of the signatures are shown

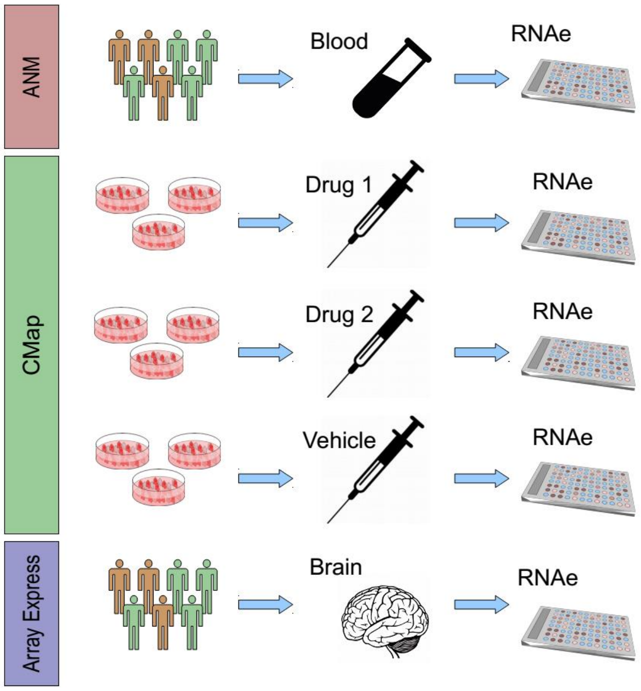


Figure 3, experimental data.

Esquematic representation of the used experimental platforms. ANM mesaures RNA expression from blood samples obtained from heathly participants and AD patients. CMap measures RNA expression from 3 different lines of immortalised human cells exposed to a collection of drugs and appropriate vehicles. Array Express measures RNA expression from postmortem brain of control participants and AD patients

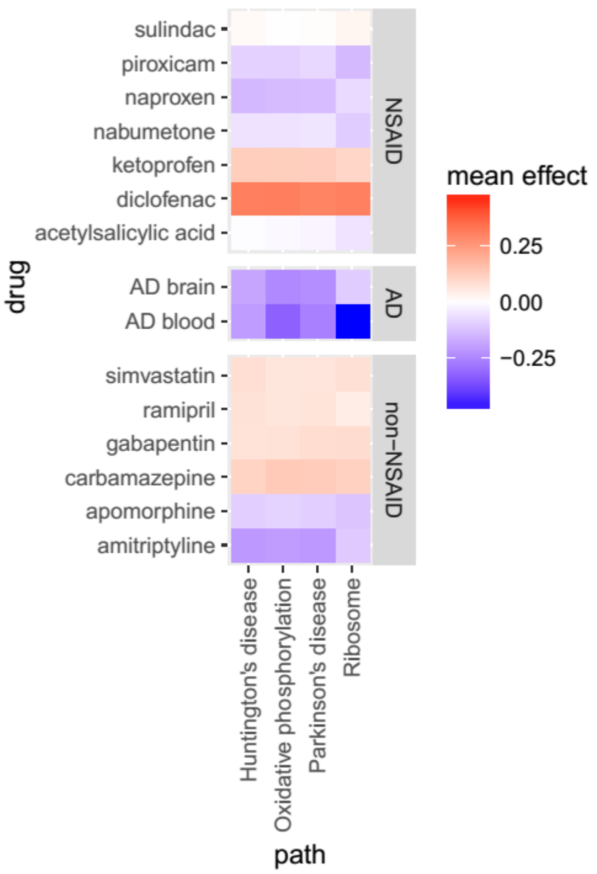


Figure 4, direction of effects:

The figure portrays the mean direction of effect of changes in gene expression for each one of the significant pathways. Rows represent the drugs or AD tissue (i.e. AD brain or AD blood) that was measured, while columns represent each one of the significant pathways. Colour is proportional to the average effect size of the genes belonging to each pathway.
